# Effect of physical activity on the change in carotid intima-media thickness: An 8-year prospective cohort study

**DOI:** 10.1371/journal.pone.0287685

**Published:** 2023-06-23

**Authors:** Byung Joon Pae, Seung Ku Lee, Soriul Kim, Ali T. Siddiquee, Yoon Ho Hwang, Min-Hee Lee, Regina E. Y. Kim, Seong Hwan Kim, Miyoung Lee, Chol Shin

**Affiliations:** 1 Institute of Human Genomic Study, College of Medicine, Korea University, Seoul, Republic of Korea; 2 Department of Pediatrics, Wayne State University School of Medicine, and the Translational Imaging Laboratory, Children’s Hospital of Michigan, Detroit, MI, United States of America; 3 Department of Cardiology, Korea University Ansan Hospital, Ansan, Republic of Korea; 4 College of Physical Education and Sport Science, Kookmin University, Seoul, Republic of Korea; 5 Biomedical Research Center, Korea University Ansan Hospital, Ansan, Republic of Korea; Radiation Effects Research Foundation, JAPAN

## Abstract

**Background and aims:**

There is a demand for longitudinal studies that use both objective and subjective measures of physical activity to investigate the association of physical activity with the change in carotid intima-media thickness (CIMT). In order to investigate such association, we conducted an 8-year follow-up study that used both objective and subjective measures of physical activity.

**Methods:**

This cohort study used subsamples of the ongoing Korean Genome and Epidemiology Study (KoGES). Included participants were between 49 to 79 years of age at baseline. Exclusion criteria included incomplete assessments of pedometer/accelerometer, international physical activity questionnaire (IPAQ), and baseline CIMT. Participants with a history of cardiovascular diseases were further excluded. Linear regression models were used for the main analysis. Age differences were assessed by stratifying the participants into < 60 years and ≥ 60 years.

**Results:**

After removing excluded participants, 835 participants were included in the final analysis (age, 59.84 ± 6.53 years; 326 (39.04%) males). 453 participants were < 60 years and 382 participants were ≥ 60 years. The daily total step count was inversely associated with the percent change in overall CIMT over 8-years (β = -0.015, standard error = 0.007, *P* = 0.034). This association was present among participants in the < 60-year-old group (β = -0.026, standard error = 0.010, *P* = 0.006), but not among participants in the ≥ 60-year-old group (β = -0.010, standard error = 0.011, *P* = 0.38).

**Conclusions:**

The findings suggest that taking preemptive actions of increasing physical activity may prevent the incidence of atherosclerosis.

## Introduction

It has been suggested by a plethora of studies that the carotid intima-media thickness (CIMT) is an indicator of the incidence of atherosclerosis [1–5]. Considering that atherosclerotic events are significant contributors of cardiovascular diseases and related deaths [6], it is imperative to identify interventions that can predict and attenuate the change in CIMT over time.

Of many interventions, physical activity has emerged as a practical solution that is associated with a truncated CIMT [7–9] and prevention of cardiovascular diseases [10–13]. Furthermore, physical activity may provide other health benefits [14, 15]. Mechanisms that can potentially elucidate the beneficial effects of physical activity on CIMT include improvements in blood pressure [16], insulin sensitivity [17], lipid contents [16], body mass index, and endothelial function via an augmented nitric oxide bioavailability [18, 19]. However, regarding the augmented nitric oxide bioavailability, it has been found that the production of nitric oxide via sheer stress is attenuated among older rats, leading to age related vascular stiffness [20]. Such phenomenon implies the potential inability of older individuals to receive the benefits of physical activity on CIMT. Thus, further investigation of the effects of physical activity on CIMT among older individuals is warranted.

With the accumulation of compelling results, there is a credence regarding the association of physical activity with CIMT [7, 8, 21–27]. However, it is crucial to acknowledge that some studies are cross-sectional in nature [7, 8, 24, 25, 27], fail to incorporate devices that can provide objective measures of physical activity [7, 22–27], lack an extensive follow-up duration [21, 22], or exclusively assess a specific sample population [7, 8, 24, 25]. In order to address such limitations, we conducted an 8-year follow-up study that used both objective and subjective measures of physical activity to assess the association of physical activity with the change in overall CIMT among participants from a general population-based cohort. We hypothesized that a greater amount of physical activity will be independently associated with a decreased change in CIMT over the course of 8 years, the independent association of greater physical activity with a decreased change in CIMT over 8-years will be diminished among older individuals, and objective measures of physical activity will have a stronger association with the 8-year change in CIMT than subjective measures.

## Materials and methods

### Participants

**Fig 1:**
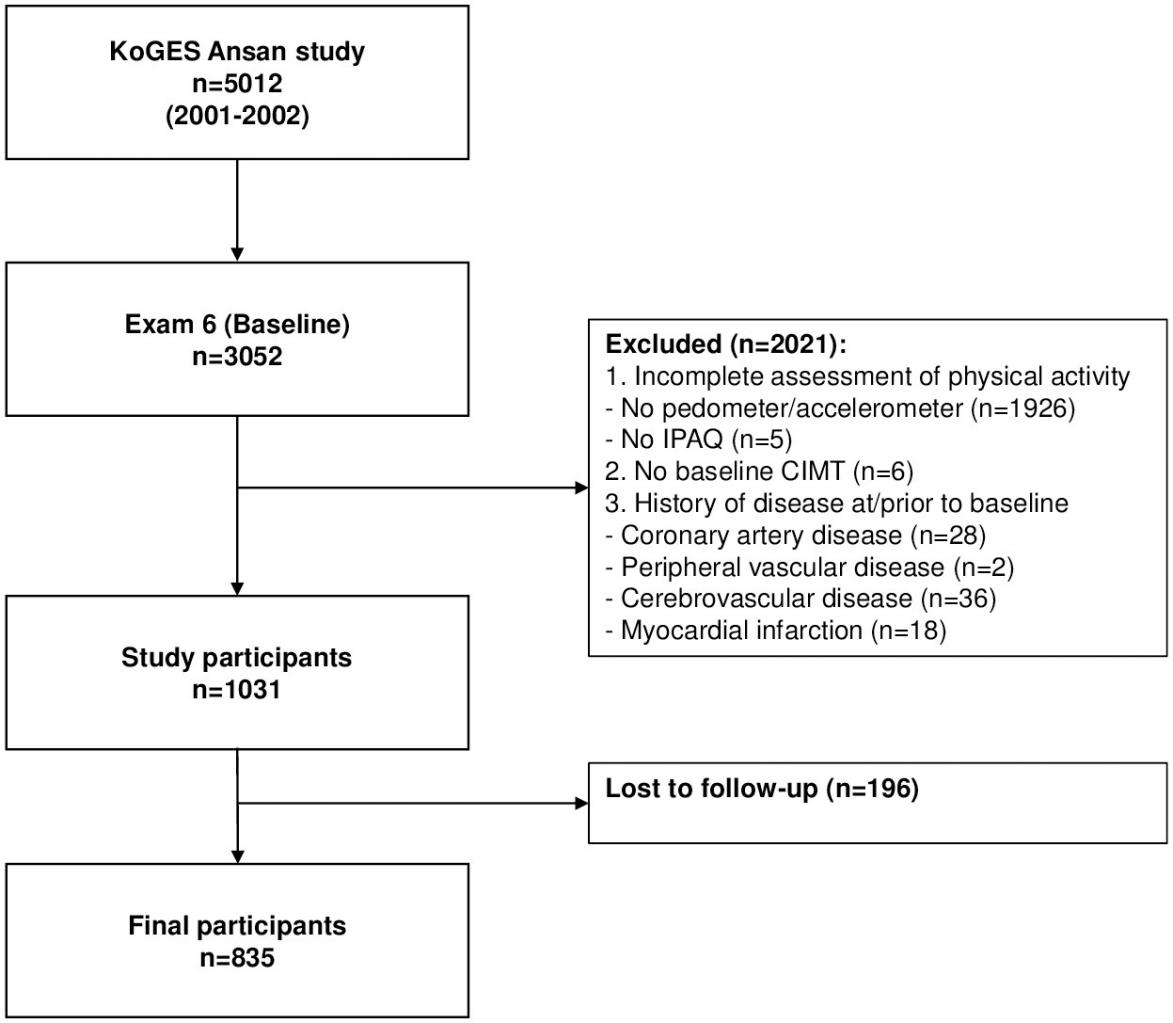
Flowchart of the recruitment of study participants. Exclusion criteria included incomplete assessments of pedometer/accelerometer (n = 1926), international physical activity questionnaire (IPAQ) (n = 5), baseline carotid intima-media thickness (CIMT) (n = 6), a history of coronary artery disease (n = 28), peripheral vascular disease (n = 2), cerebrovascular disease (n = 36), and myocardial infarction (n = 18).

Participants involved in the current study were recruited from the ongoing Korean Genome and Epidemiology Study (KoGES)-Ansan cohort, which is a prospective cohort study that had enrolled Koreans in their mid to late adulthood from a general population [28, 29]. The original Ansan cohort started in 2001, included a total of 5012 participants, and took place in Ansan, South Korea. The current study used a subsample of the ongoing cohort and was approved by the institutional review board of Korea University Ansan Hospital (IRB No. 2006AS0045). All participants provided written informed consent. Baseline assessments of physical activity, general characteristics, and CIMT were conducted between 2011–2012 (exam 6), and the follow-up assessment of CIMT was carried out between 2019–2020 (exam 10). Of the 3052 participants who showed up to the baseline assessment, 1926 participants were excluded due to missing pedometer/accelerometer data. 11 participants were excluded due to missing international physical activity questionnaire (IPAQ) (n = 5) and baseline CIMT (n = 6) data. In addition, participants with a history of diagnosis/treatment for coronary artery disease (n = 28), peripheral vascular disease (n = 2), cerebrovascular disease (n = 36), or myocardial infarction (n = 18) were excluded. 196 participants were lost to follow-up, resulting in a final sample size of 835 (Fig 1).

### Carotid intima-media thickness

The assessment of CIMT was carried out by utilizing B mode ultrasonography (baseline: Titan, Sonosite, Bothell, WA, USA; follow-up: MicroMaxx, Sonosite, Bothell, WA, USA). The two devices were validated. The distal ends of the far and near walls of the left and right common carotid arteries were measured, and measurements were obtained approximately 1cm away from the bifurcation point [30, 31]. The overall CIMT was calculated by averaging the mean thickness values of the far and near walls of the left and right common carotid arteries. The dependent variable, change in overall CIMT, was expressed as a percentage (ΔCIMT (%) = 100 × [(follow-up thickness–baseline thickness) / baseline thickness]) in order to accommodate for the differences in baseline overall CIMT.

### Accelerometer

At baseline, participants of the current study were instructed to wear a tri-axial, belt accelerometer (Actigraph GT3X, Actigraph Corporation, Pensacola, FL, USA) for 7 consecutive days. The accelerometer was not worn while participants were sleeping/swimming/showering, and the epoch time was set to 60 seconds. The daily total amount of physical activity, expressed as minutes/day, was calculated by averaging the results over the 7-day period. Within the daily total amount of physical activity, 6 different subsections were categorized by the software (ActiLife Version 5.10.0, Actigraph Corporation, Pensacola, FL, USA) based on intensity levels: Sedentary (0–100 counts/minute), Lifestyle (101–759 counts/minute), Light (760–1952 counts/minute), Moderate (1953–5724 counts/minute), Vigorous (5725–9498 counts/minute), and Very Vigorous (equal to or greater than 9499 counts/minute). Estimates of metabolic equivalent of task (MET) can be calculated from the counts/minute obtained by a tri-axial accelerometer [32, 33]. Due to 0 counts/minute being registered as sedentary activity and sedentary activity being the predominant contributor of the daily total, we used the sum of moderate, vigorous, and very vigorous activity minutes (actigraph-MVV) as well as the percentage of moderate, vigorous, and very vigorous activity minutes (actigraph-percent of MVV) for the regression analysis.

### Pedometer

In addition to the accelerometer, participants were told to simultaneously wear a belt pedometer (Omron HJ-720ITC, Omron Healthcare, Kyoto, Japan) for the same 7-day period. As with the accelerometer, the pedometer was not worn while the participants were sleeping/swimming/showering. The pedometer automatically registered aerobic steps if the speed was at least 60 steps per minute and the ambulatory activity was carried out for more than 10 consecutive minutes using the Omron Health Management Software. The total number of steps per day and the total number of aerobic steps per day were obtained from the pedometer.

### International Physical Activity Questionnaire (IPAQ)-long form

For the subjective measure of physical activity, participants completed the IPAQ-long form on the same day that they were assessed for baseline CIMT and general characteristics. The IPAQ-long form consists of 4 different sections: Work, Transportation, Domestic, and Leisure. Each section assesses the subjective amount of low, moderate, and vigorous physical activity time during the past 7 days. The amount of physical activity time during both weekdays and weekend days were obtained and weighted based on the number of week/weekend days in a week. The total score was calculated by summing the 4 section totals and expressed as a continuous variable in the units of MET-min/week. Further details can be found elsewhere (www.ipaq.ki.se).

### Covariates

On the same day of baseline CIMT assessment, data regarding general characteristics such as age, sex, smoking status, drinking status, presence of hypertension/type 2 diabetes, body mass index, lipid profiles, education, and income were collected. Total cholesterol, high-density lipoprotein cholesterol, triglycerides, and glucose levels were measured via an analyzer (ADVIA 1800, Bayer, Germany) following an 8-hour fast and expressed in the unit of mg/dL. Hypertension was defined as having a history of medication use/systolic blood pressure of 140 mmHg or greater/diastolic blood pressure of 90 mmHg or greater while type 2 diabetes was defined as having a history of medication use/diagnosis/fasting blood sugar level of 126 mg/dL or greater/2hr blood sugar level of 200 mg/dL or greater. 17 participants were missing data regarding type 2 diabetes, and 15 were missing income data. Both smoking and drinking were coded into never vs. ever. The height (m) and weight (kg) were used to calculate the body mass index (kg/m^2^), and the Friedewald equation was used to calculate low-density lipoprotein cholesterol [34].

### Statistical analysis

All statistical analyses were completed by using IBM SPSS Statistics version 26.0 for Windows (IBM Corp, Armonk, NY, USA). Regarding the comparisons of general characteristics between the two age groups, continuous variables were compared using the Student’s *t* test or Mann-Whitney U test, and categorical variables were compared using the χ^2^ test. In order to assess whether the percent change in overall CIMT over the 8-year period was significant, we conducted the paired *t* test. Linear regression models were utilized to investigate the independent association of baseline physical activity with the percent change in overall CIMT over 8 years. The fully adjusted model included age, sex, smoking status, drinking status, presence of hypertension, presence of type 2 diabetes, body mass index, and total cholesterol as covariates. Natural log transformations were conducted for data manifesting non-normality. Each physical activity parameter involved in the regression analysis was tested separately, and all predictors included in regression models were of baseline values. Furthermore, the same regression models were conducted among participants less than 60 years of age and participants equal to or greater than 60 years of age in order to scrutinize the age differences. Statistical significance was determined when *P* < 0.05.

## Results

### General characteristics

Table 1 displays the baseline general characteristics of the participants. Of the total participants (n = 835) (age, 59.84 ± 6.53 years; 326 males (39.04%)), 453 were less than 60 years of age and 382 were equal to or greater than 60 years of age. The age < 60 group had a higher total cholesterol content (202.45 ± 34.05 mg/dL vs. 194.71 ± 36.70 mg/dL; *P* = 0.002), low-density lipoprotein cholesterol content (124.15 ± 32.67 mg/dL vs. 118.82 ± 34.17 mg/dL; *P* = 0.02), and more ever drinkers compared to the age ≥ 60 group (48.12% vs. 40.31%; *P* = 0.024). However, the age ≥ 60 group was older (65.50 ± 4.84 years vs. 55.06 ± 2.99 years; *P* < 0.001), had a greater overall CIMT (0.78 ± 0.08 mm vs. 0.73 ± 0.07 mm; *P* < 0.001), total number of aerobic steps (median [IQR], 1378.43 [392.57–3176.14] steps/day vs. 917.57 [170.36–2852.07] steps/day; *P* = 0.002), prevalence of hypertension (46.34% vs. 27.59%; *P* < 0.001), and prevalence of type 2 diabetes (24.20% vs. 13.80%; *P* < 0.001).

**Table 1:** General characteristics at baseline.

		All participants (n = 835)	Age < 60 yrs (n = 453)	Age ≥ 60 yrs (n = 382)	*P*-value^a^
Age, y		59.84 (6.53)	55.06 (2.99)	65.50 (4.84)	<0.001
Males		326 (39.04)	168 (37.09)	158 (41.36)	0.21
Ever smokers		265 (31.74)	138 (30.46)	127 (33.25)	0.39
Ever drinkers		372 (44.55)	218 (48.12)	154 (40.31)	0.024
Hypertension		302 (36.17)	125 (27.59)	177 (46.34)	<0.001
Type 2 diabetes		152 (18.58)	61 (13.80)	91 (24.20)	<0.001
BMI, kg/m^2^		24.58 (2.85)	24.59 (2.80)	24.58 (2.90)	0.94
Total cholesterol, mg/dL		198.91 (35.48)	202.45 (34.05)	194.71 (36.70)	0.002
LDL cholesterol, mg/dL		121.71 (33.45)	124.15 (32.67)	118.82 (34.17)	0.02
HDL cholesterol, mg/dL		49.37 (12.74)	50.04 (12.76)	48.57 (12.69)	0.10
Triglyceride, mg/dL		139.15 (92.63)	141.29 (97.17)	136.61 (87.00)	0.47
Education					<0.001
Primary education		204 (24.43)	74 (16.33)	130 (34.03)	
Secondary education		508 (60.84)	309 (68.21)	199 (52.09)	
Tertiary education		123 (14.73)	70 (15.45)	53 (13.87)	
Income					<0.001
< 3,000,000 won		438 (53.41)	177 (39.60)	261 (69.97)	
≥ 3,000,000 won		382 (46.59)	270 (60.40)	112 (30.03)	
**Carotid Intima-Media Thickness**					
Overall CIMT, mm		0.75 (0.08)	0.73 (0.07)	0.78 (0.08)	<0.001
Right far wall, mm		0.74 (0.10)	0.72 (0.10)	0.76 (0.10)	<0.001
Left far wall, mm		0.74 (0.11)	0.72 (0.10)	0.76 (0.11)	<0.001
Right near wall, mm		0.76 (0.10)	0.73 (0.10)	0.79 (0.10)	<0.001
Left near wall, mm		0.76 (0.10)	0.74 (0.09)	0.79 (0.10)	<0.001
**Physical Activity**					
Omron-Total steps, steps/day	mean (SD)	8155.66 (3524.79)	8157.32 (3536.44)	8153.70 (3515.57)	0.99^c^
	median (IQR)	7516.60 (5633.50–10424.57)	7583.17 (5583.11–10401.45)	7457.43 (5631.89–10507.04)	0.95^d^
Omron-Aerobic steps, steps/day	mean (SD)	1993.10 (2274.02)	1821.96 (2189.32)	2196.05 (2357.36)	0.02^c^
	median (IQR)	1144.71 (253.86–2997.00)	917.57 (170.36–2852.07)	1378.43 (392.57–3176.14)	0.002^d^
Actigraph-MVV, min/day	mean (SD)	40.70 (32.44)	40.21 (26.84)	41.27 (38.06)	0.64^c^
	median (IQR)	34.86 (18.71–55.33)	35.71 (19.50–55.14)	34.29 (17.79–55.93)	0.78^d^
Actigraph-Percent of MVV, %^b^	mean (SD)	4.33 (3.32)	4.32 (2.86)	4.35 (3.79)	0.87^c^
	median (IQR)	3.70 (2.05–5.91)	3.74 (2.12–5.84)	3.62 (1.98–5.94)	0.60^d^

Data are presented as n (%) for categorical variables and mean (SD) for continuous variables unless otherwise stated.

BMI, body mass index; LDL, low-density lipoprotein cholesterol; HDL, high-density lipoprotein cholesterol; CIMT, carotid intima-media thickness; MVV, moderate to very vigorous.

SI conversion: To convert cholesterol to millimoles per liter, multiply by 0.0259; to convert triglycerides to millimoles per liter, multiply by 0.0113.

^a^ Comparison between the two age groups.

^b^ Expressed as percentage of time spent performing MVV per day.

^c^ Compared using Student’s *t* test.

^d^ Compared using Mann-Whitney U test.

### Change in carotid intima-media thickness during the 8-year follow-up

**Fig 2:**
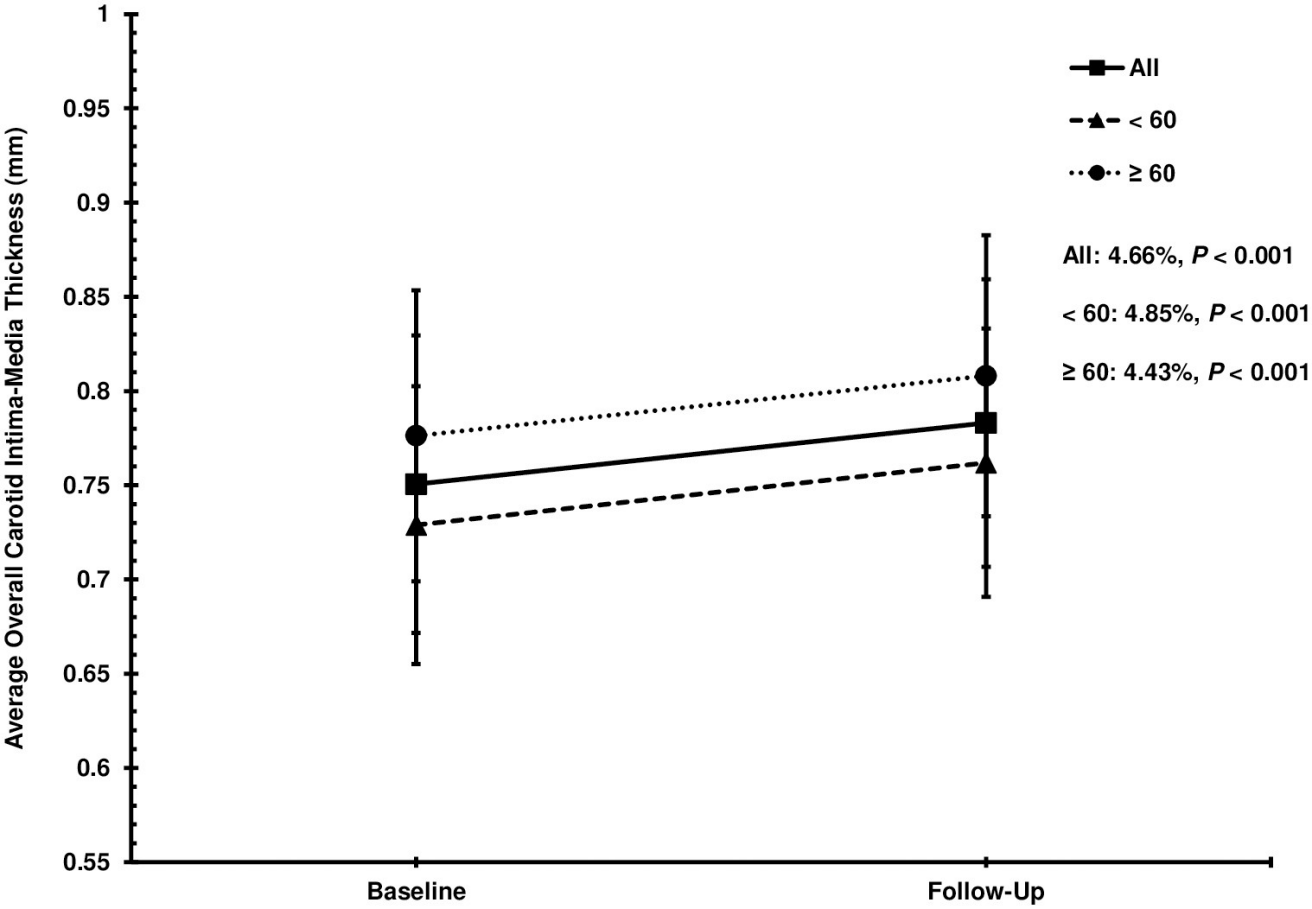
Average overall carotid intima-media thickness (CIMT) values. Baseline and follow-up values of the average overall carotid intima-media thickness (CIMT) for all participants (closed squares, n = 835), participants less than 60 yrs of age (closed triangles, n = 453), and participants equal to or greater than 60 yrs of age (closed circles, n = 382). Displayed percentages represent the average percent change in overall carotid intima-media thickness from baseline to follow-up. The results of the paired *t* test are delineated by the *P* values. Data are unadjusted and expressed as mean ± SD.

The total participants and age-stratified groups experienced increases in the overall CIMT relative to the baseline thickness during the 8-year period (all, 4.66%; age < 60, 4.85%; age ≥ 60, 4.43%) (Fig 2). The results of the paired *t* test corroborate for the significance of such increases (all at baseline, 0.75 ± 0.08 mm and follow-up, 0.78 ± 0.08 mm, *P* < 0.001; age < 60 at baseline, 0.73 ± 0.07 mm and follow-up, 0.76 ± 0.07 mm, *P* < 0.001; age ≥ 60 at baseline, 0.78 ± 0.08 mm and follow-up, 0.81 ± 0.07 mm, *P* < 0.001). The greatest percent increase in overall CIMT was observed in the age < 60 group while the smallest was observed in the age ≥ 60 group (4.85% vs. 4.43%, respectively) (Fig 2). However, the age ≥ 60 group manifested the thickest overall CIMT at both baseline and follow-up (overall CIMT at baseline, 0.78 ± 0.08 mm and follow-up, 0.81 ± 0.07 mm) (Fig 2).

### Association of physical activity with the change in carotid intima-media thickness

The results of the linear regression analysis using total steps as the main predictor are shown in Table 2. The total number of steps per day was inversely associated with the percent change in overall CIMT over 8-years for all participants (n = 835) (model 1: β (SE) = -0.017 (0.007), *P* = 0.017; model 2: β (SE) = -0.015 (0.007), *P* = 0.034). However, aerobic steps and the accelerometer-based physical activity parameters were not associated with the percent change in overall CIMT (Table 3). Moreover, the IPAQ score was not associated with the percent change in overall CIMT over 8-years (model 1: β (SE) = 0.103 (0.135), *P* = 0.45; model 2: β (SE) = 0.074 (0.135), *P* = 0.58) (S1 Table in S1 File).

**Table 2:** Association of total steps with percent change in overall carotid intima-media thickness.

	Model 1^a^			Model 2^b^		
All (n = 835)	β	SE	*P*-Value	β	SE	*P*-Value
Omron-Total steps, 100 steps/day	-0.017	0.007	0.017	-0.015	0.007	0.034
**Age < 60 (n = 453)**						
Omron-Total steps, 100 steps/day	-0.022	0.010	0.022	-0.026	0.010	0.006
**Age ≥ 60 (n = 382)**						
Omron-Total steps, 100 steps/day	-0.011	0.011	0.29	-0.010	0.011	0.38

^a^ Unadjusted model.

^b^ Adjusted for age, sex, smoking, drinking, hypertension, type 2 diabetes, body mass index, and total cholesterol.

**Table 3:** Association of objective physical activity parameters with percent change in overall carotid intima-media thickness among different age groups.

	Model 1^a^			Model 2^b^		
All (n = 835)	β	SE	*P*-Value	β	SE	*P*-Value
Omron-Aerobic steps, 100 steps/day^c^	-0.280	0.183	0.13	-0.188	0.184	0.31
Actigraph-MVV, 10 min/day^c^	-0.334	0.306	0.28	-0.301	0.318	0.34
Actigraph-Percent of MVV, %^c^	-0.249	0.309	0.42	-0.217	0.320	0.50
**Age < 60 (n = 453)**						
Omron-Aerobic steps, 100 steps/day^c^	-0.572	0.237	0.016	-0.656	0.235	0.005
Actigraph-MVV, 10 min/day^c^	-0.936	0.435	0.032	-1.160	0.432	0.007
Actigraph-Percent of MVV, %^c^	-0.886	0.441	0.044	-1.081	0.437	0.013
**Age ≥ 60 (n = 382)**						
Omron-Aerobic steps, 100 steps/day^c^	0.162	0.289	0.57	0.256	0.292	0.38
Actigraph-MVV, 10 min/day^c^	0.210	0.431	0.63	0.198	0.477	0.68
Actigraph-Percent of MVV, %^c^	0.312	0.432	0.47	0.293	0.474	0.54

MVV, moderate to very vigorous.

^a^ Unadjusted model.

^b^ Adjusted for age, sex, smoking, drinking, hypertension, type 2 diabetes, body mass index, and total cholesterol.

^c^ Data were log-transformed.

### Moderating effect of age

In the age < 60 group, the total number of steps per day was inversely associated with the percent change in overall CIMT over 8-years (model 1: β (SE) = -0.022 (0.010), *P* = 0.022; model 2: β (SE) = -0.026 (0.010), *P* = 0.006) (Table 2). In contrast, there was no independent association of total number of steps per day with the percent change in overall CIMT over 8-years in the age ≥ 60 group (model 1: β (SE) = -0.011 (0.011), *P* = 0.29; model 2: β (SE) = -0.010 (0.011), *P* = 0.38) (Table 2).

When using other objective measures of physical activity as main predictors (i.e. aerobic steps, actigraph-MVV, and actigraph-percent of MVV), all parameters were inversely associated with the percent change in overall CIMT in the age < 60 group after adjusting for covariates (aerobic steps, β (SE) = -0.656 (0.235), *P* = 0.005; actigraph-MVV, β (SE) = -1.160 (0.432), *P* = 0.007; actigraph-percent of MVV, β (SE) = -1.081 (0.437), *P* = 0.013). However, such association was diminished in the age ≥ 60 group (Table 3). No difference was observed between the two age groups regarding the association of IPAQ score with the percent change in overall CIMT over 8-years. (S1 Table in S1 File).

## Discussion

The findings of this study have allowed us to identify the relationship between physical activity and CIMT, in which physical activity was associated with a reduced progression of CIMT. In the present study, a higher amount of physical activity, specifically the total number of steps, was independently associated with a reduced change in overall CIMT over 8-years. Moreover, this association was clearly observed among participants under 60 years of age. However, the subjective measure of physical activity was not associated with the percent change in overall CIMT over the 8-year follow-up.

Our findings provide clinical implications that suggest physical activity may reduce the risk of an atherosclerotic event, as 100 additional total steps taken per day at baseline led to a 0.015% decreased change in overall CIMT over 8-years. Furthermore, our findings imply that physical activity may slow down the age related progression of CIMT. Age is known to be a potent risk factor for various diseases [35–40], and thickening of the CIMT is no exception [41–44]. Considering that the thickest overall CIMT observed from any group in the current study was 0.81 ± 0.07 mm, which was exhibited by the age ≥ 60 group at follow-up, and that it is under the cut-off for abnormal CIMT [45, 46], it can be concluded that the participants of this study have healthy vasculatures. Despite having healthy vasculatures, on average, all participants experienced significant increases in overall CIMT relative to baseline. However, with 100 additional total steps/day at baseline, the progression of CIMT was attenuated by 0.015%. This decrease of 0.015% may not be sufficient enough to nullify the age related thickening of CIMT, but it can slow down the process. Therefore, it could be understood that increased physical activity may slow down the age related thickening of CIMT. Mechanisms such as improved blood pressure [16], insulin sensitivity [17], lipid contents [16], body mass index, and endothelial function via an augmented nitric oxide bioavailability [18, 19] could explain the association of baseline physical activity with the percent change in overall CIMT over 8-years.

Regarding the age differences, the inverse association of baseline physical activity with the percent change in overall CIMT over 8-years was present in the age < 60 group but not in the age ≥ 60 group. For the age < 60 group, an increase of 100 total steps per day at baseline led to a 0.026% decrease in the 8-year change in overall CIMT, which was greater than it was for all participants (-0.026% vs. -0.015%, respectively). Furthermore, the other 3 objective measures of physical activity (i.e. aerobic steps, actigraph-MVV, and actigraph-percent of MVV) were also inversely associated with the percent change in overall CIMT over 8-years in the age < 60 group but not in the age ≥ 60 group. This highlights the discrepancy in results between the two age groups. It is imperative to acknowledge that, from a statistical standpoint, such discrepancy in results between the two age groups could be explained by the difference in sample size and prevalence of categorical variables such as drinking, hypertension, and type 2 diabetes. However, physiological mechanisms could also elucidate this finding. The age < 60 group of the current study experienced an average increase of 4.85% in the overall CIMT over 8-years while the age ≥ 60 group experienced a 4.43% average increase. Despite the lack of statistical significance regarding the difference in average change in CIMT over 8-years (4.85% vs. 4.43%), this result supports the idea that the vasculatures of older individuals are susceptible to stiffening [47–50]. It could be speculated that the lack of association of baseline physical activity with the 8-year percent change in overall CIMT in the age ≥ 60 group can be attributed to stiffened vasculatures.

In this study, the pedometer-based total step count was inversely associated with the 8-year percent change in overall CIMT. For participants under 60 yrs, both pedometer and accelerometer-based physical activity measures were inversely associated with the 8-year change in overall CIMT. Particularly, the accelerometer results among participants under 60 yrs suggest the notable effectiveness of accelerometer-based moderate to very vigorous physical activity for CIMT. The IPAQ score was not associated with the percent change in overall CIMT over 8-years in our results, potentially questioning the validity of subjective measures of physical activity. Despite the lack of association of IPAQ scores with the percent change in overall CIMT over 8-years, it is of interest to recognize that repeated measures of the IPAQ score could ameliorate this lack of association.

The current study has several limitations that must be addressed. The observational nature of the current study implies that potential bias stemming from non-randomization could have been present, as participants who were able to conduct more ambulatory activity may have been healthier [51]. The large number of excluded participants could have introduced bias regarding the sampling process, also resulting in a small sample size. However, the excluded participants were generally healthier regarding the clinical variables, suggesting that random sampling was ensured (S2 Table in S1 File). The loss of 1926 participants can be attributed to the fragility and price of the pedometer/accelerometers. In addition, the current study did not assess cardiorespiratory fitness, which may be more important than the amount of physical activity [52–56]. Future work should attempt to incorporate randomization, assess variables related to cardiorespiratory fitness (i.e. VO_2_ max), increase the sample size, and additionally implement data regarding persistent physical activity behaviors to investigate whether persistent physical activity can confer augmented health benefits.

## Conclusion

The current study has demonstrated that greater amounts of physical activity are associated with a decreased percent change in overall CIMT over 8-years, but not among participants equal to or greater than 60 years of age. Early lifestyle modification, particularly increasing the amount of physical activity, may prevent the development of atherosclerosis.

## Supporting information

S1 File (DOCX)
