# Supplementary material for: Effect of physical activity on the change in carotid intima-media thickness: An 8-year prospective cohort study
Source: PLoS One. 2023 Jun 23;18(6):e0287685. doi: 10.1371/journal.pone.0287685 (PMC10289364; doi:10.1371/journal.pone.0287685)
Supplement: S1 File — (DOCX) [file pone.0287685.s001.docx]

**Supporting Information**

**Effect of Physical Activity on the Change in Carotid Intima-Media Thickness: an 8-Year Prospective Cohort Study**

Byung Joon Pae^1^, Seung Ku Lee^1^, Soriul Kim^1^, Ali T. Siddiquee^1^, Yoon Ho Hwang^1^, Min-Hee Lee^1,2^, Regina E. Y. Kim^1^, Seong Hwan Kim^3^, Miyoung Lee^4^, Chol Shin^1,5^

^1^ Institute of Human Genomic Study, College of Medicine, Korea University, Seoul, Republic of Korea

^2^ Department of Pediatrics, Wayne State University School of Medicine, and the Translational Imaging Laboratory, Children’s Hospital of Michigan, Detroit, MI, USA

^3^ Department of Cardiology, Korea University Ansan Hospital, Ansan, Republic of Korea

^4^ College of Physical Education and Sport Science, Kookmin University, Seoul, Republic of Korea

^5^ Biomedical Research Center, Korea University Ansan Hospital, Ansan, Republic of Korea

**S1 Table**. Association of International Physical Activity Questionnaire (IPAQ) with Percent Change in Overall Carotid Intima-Media Thickness

|  | **Model 1**^a^ | | | **Model 2**^b^ | | |
| --- | --- | --- | --- | --- | --- | --- |
| **All** | **β** | **SE** | ***P*-Value** | **β** | **SE** | ***P*-Value** |
| IPAQ, MET*min/week^c^ | 0.103 | 0.135 | 0.45 | 0.074 | 0.135 | 0.58 |
| **Age < 60** |  |  |  |  |  |  |
| IPAQ, MET*min/week^c^ | 0.120 | 0.166 | 0.47 | 0.059 | 0.163 | 0.72 |
| **Age ≥ 60** |  |  |  |  |  |  |
| IPAQ, MET*min/week^c^ | 0.092 | 0.232 | 0.69 | 0.130 | 0.232 | 0.57 |

MET, metabolic equivalent of task.

^a^ Unadjusted model.

^b^ Adjusted for age, sex, smoking, drinking, hypertension, type 2 diabetes, body mass index, and total cholesterol.

^c^ Data were log-transformed.

**S2 Table**. Differences in Baseline General Characteristics Between Included and Excluded Participants

|  | **Included participants (n=835)** | **Excluded participants (n=1803)**^a^ | ***P*-value** |
| --- | --- | --- | --- |
| Age, y | 59.84 (6.53) | 56.19 (6.34) | <0.001 |
| Males | 326 (39.04) | 986 (54.69) | <0.001 |
| Ever smokers | 265 (31.74) | 767 (42.54) | <0.001 |
| Ever drinkers | 372 (44.55) | 1014 (56.24) | <0.001 |
| Hypertension | 302 (36.17) | 575 (31.89) | 0.03 |
| Type 2 diabetes^b^ | 152 (18.58) | 306 (17.59) | 0.54 |
| BMI, kg/m^2^ | 24.58 (2.85) | 24.68 (2.97) | 0.43 |
| Total cholesterol, mg/dL | 198.91 (35.48) | 199.23 (34.58) | 0.83 |
| LDL cholesterol, mg/dL | 121.71 (33.45) | 122.54 (31.89) | 0.54 |
| HDL cholesterol, mg/dL | 49.37 (12.74) | 48.48 (12.02) | 0.08 |
| Triglyceride, mg/dL | 139.15 (92.63) | 141.06 (85.79) | 0.60 |
| Education |  |  | <0.001 |
| Primary education | 204 (24.43) | 288 (15.97) |  |
| Secondary education | 508 (60.84) | 1126 (62.45) |  |
| Tertiary education | 123 (14.73) | 389 (21.58) |  |
| Income^c^ |  |  | <0.001 |
| < 3,000,000 won | 438 (53.41) | 728 (40.92) |  |
| ≥ 3,000,000 won | 382 (46.59) | 1051 (59.08) |  |
| **Carotid Intima-Media Thickness** |  |  |  |
| Overall CIMT, mm^d^ | 0.75 (0.08) | 0.74 (0.07) | <0.001 |
| Right far wall, mm | 0.74 (0.10) | 0.74 (0.10) | 0.17 |
| Left far wall, mm | 0.74 (0.11) | 0.73 (0.10) | 0.06 |
| Right near wall, mm | 0.76 (0.10) | 0.75 (0.10) | 0.013 |
| Left near wall, mm | 0.76 (0.10) | 0.74 (0.09) | <0.001 |

Data are presented as n (%) for categorical variables and mean (SD) for continuous variables unless otherwise stated.

BMI, body mass index; LDL, low-density lipoprotein cholesterol; HDL, high-density lipoprotein cholesterol; CIMT, carotid intima-media thickness; MVV, moderate to very vigorous.

SI conversion: To convert cholesterol to millimoles per liter, multiply by 0.0259; to convert triglycerides to millimoles per liter, multiply by 0.0113.

^a^ Participants who were excluded due to missing pedometer/accelerometer data (n=1926) and also free of cardiovascular diseases (coronary artery disease, n=40; peripheral vascular disease, n=8; cerebrovascular disease, n=50; myocardial infarction, n=24; congestive heart failure, n=1).

^b^ 63 of the 1803 participants were missing data regarding type 2 diabetes.

^c^ 24 of the 1803 participants were missing data regarding income.

^d^ 19 of the 1803 participants were missing data regarding overall CIMT.
